# Mindfulness-based neurofeedback: A systematic review of EEG and fMRI studies

**DOI:** 10.1162/imag_a_00396

**Published:** 2024-12-20

**Authors:** Isaac N. Treves, Keara D. Greene, Zia Bajwa, Emma Wool, Nayoung Kim, Clemens C.C. Bauer, Paul A. Bloom, David Pagliaccio, Jiahe Zhang, Susan Whitfield-Gabrieli, Randy P. Auerbach

**Affiliations:** Department of Brain and Cognitive Sciences, Massachusetts Institute of Technology Cambridge, MA, United States; Department of Psychology, Northeastern University, Boston, MA, United States; Center for Precision Psychiatry, Department of Psychiatry, Massachusetts General Hospital, Boston, MA, United States; Department of Psychiatry, Columbia University, New York, NY, United States; Division of Child and Adolescent Psychiatry, New York State Psychiatric Institute, New York, NY, United States; Northeastern University Biomedical Imaging Center, Boston, MA, United States

**Keywords:** neurofeedback, mindfulness, EEG, fMRI, DMN, theta

## Abstract

Neurofeedback concurrent with mindfulness meditation may reveal meditation effects on the brain and facilitate improved mental health outcomes. Here, we systematically reviewed electroencephalography (EEG) and functional magnetic resonance imaging (fMRI) studies of mindfulness meditation with neurofeedback (mbNF) and followed PRISMA guidelines. We identified 9 fMRI reports, consisting of 177 unique participants, and 9 EEG reports, consisting of 242 participants. Studies of fMRI focused primarily on downregulating the default-mode network (DMN). Although studies found decreases in DMN activations during neurofeedback, there is a lack of evidence for transfer effects, and the majority of studies did not employ adequate controls, for example, sham neurofeedback. Accordingly, DMN decreases may have been confounded by general task-related deactivation. EEG studies typically examined alpha, gamma, and theta frequency bands, with the most robust evidence supporting the modulation of theta band activity. Both EEG and fMRI mbNF have been implemented with high fidelity in clinical populations. However, the mental health benefits of mbNF have not been established. In general, mbNF studies would benefit from sham-controlled RCTs, as well as clear reporting (e.g., CRED-NF).

## 1 Introduction

Mindfulness meditation involves cultivating an accepting, open-minded attention to the present moment (Creswell, 2017). The word mindfulness originated from Eastern contemplative traditions, specifically, as a translation of the term*sati*from Pali or*smrti*from Sanskrit, which mean remembering or being aware. Mindfulness was largely introduced to Western medicine with the advent of mindfulness-based stress reduction (MBSR) in the 1980s (Kabat-Zinn, 1982,^2003^). MBSR and its adaptations have been used to treat chronic pain (Goyal et al., 2014;Kabat-Zinn, 1982), anxiety (Goldin et al., 2013;Hoge et al., 2023;Hölzel et al., 2013), addiction (Black, 2014;Garland et al., 2015;Vallejo & Amaro, 2009), and depression (Kuyken et al., 2016). Indeed, mindfulness has been documented to be equally effective as pharmacological treatment for anxiety disorders (Hoge et al., 2023) and potentially more effective than cognitive behavioral therapy (CBT) for treatment of mild-to-moderate depression (Strauss et al., 2023). Mindfulness is now a central component of leading psychotherapeutic approaches such as dialectical behavioral therapy (DBT) (Linehan, 1993;McCauley et al., 2018) and acceptance and commitment therapy (ACT) (Hayes et al., 1999).

Mindfulness meditations include practices such as*breath awareness*, which involves orienting attention to one’s breath and practicing returning to the breath every time one’s attention wanders away, and*body scans*, involving moving the spotlight of attention from body part to body part with a curious and non-judgmental attitude toward the sensations one encounters. Another practice is*open monitoring*, where one notices transient thoughts and sensations in an open state without attaching to them. Breath awareness and body scans are often called*focused attention*(FA) practices, aiming to cultivate a stable and precise attention, which contrasts with*open monitoring*(OM) practices, cultivating receptivity to experience (Lutz et al., 2008).

**Fig. 1.**
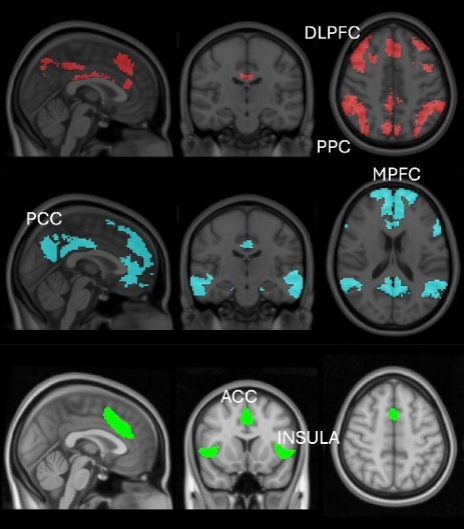
Brain networks involved in mindfulness meditation. Central executive network, in red; default-mode network, in blue; salience network, in green. DLPFC: dorsolateral prefrontal cortex; PPC: posterior parietal cortex; PCC: posterior cingulate cortex; MPFC: medial prefrontal cortex; ACC: anterior cingulate cortex; insula: insular cortex. Adapted with permission fromTreves et al. (in press).

**Fig. 2.**
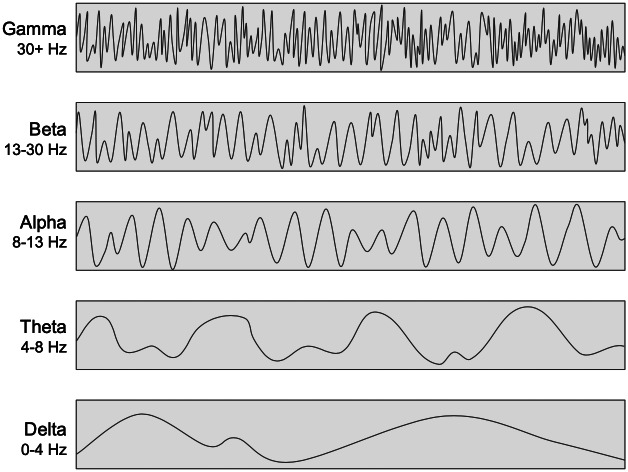
EEG frequency bands. This visualization demonstrates the differing frequencies of the various EEG bands. Created in Google slides.

There are several theories regarding the neurobiological mechanisms behind mindfulness meditation. One influential account suggests that large-scale brain networks are involved (Hasenkamp et al., 2012;Mooneyham et al., 2016). Specifically, this account implicates the default-mode network (DMN), with core regions of the posterior cingulate cortex (PCC) and medial prefrontal cortex (mPFC) as well as the central executive network (CEN), with core regions of the dorsolateral prefrontal cortex (DLPFC) and parietal cortex, and the salience network (SN), with core regions of the anterior cingulate cortex (ACC) and insula (Fig. 1). In this account, the DMN is involved in mind-wandering away from the object of meditation, the CEN is involved in goal-directed maintenance of the object, and the SN is involved in switching between the two (Hasenkamp et al., 2012). This is based largely on functional magnetic resonance imaging (fMRI) during focused attention meditation (Fox et al., 2016;Ganesan et al., 2022), changes observed in networks after mindfulness training (Rahrig et al., 2022;Sezer et al., 2022), in addition to a robust cognitive neuroscience literature on these networks (Menon, 2011). In tandem, researchers have examined changes in brain rhythms or oscillations during meditation using electroencephalography (EEG) (Fig. 2). Brain oscillations represent information processing across wide-ranging brain regions, and change with attention (Herrmann et al., 2016). There is evidence of power increases in alpha, theta, and gamma waves during meditation (Chiesa & Serretti, 2010;Lee et al., 2018;Lomas et al., 2015;Stapleton et al., 2020). Alpha and theta power may correspond to inwardly focused attention (Lomas et al., 2015), whereas gamma power may reflect broad awareness (Lomas et al., 2015;Lutz et al., 2004;Stapleton et al., 2020). Despite this meaningful work, the field still lacks a complete mechanistic account of mindfulness meditation. Take, for example, the causal model that mindfulness meditation decreases DMN activation (Brewer et al., 2011;Ganesan et al., 2022). This particular model is largely founded on task-based fMRI research comparing meditation with control conditions. Task-based fMRI leads to great insight into brain regions and networks associated with meditation, but cannot directly show mechanistic involvement. For example, neural changes associated with mindfulness may be caused by decreases in stress accompanying meditation, rather than the voluntary and directed actions of meditation. In addition, the choice of control condition can lead to differing results. For example,Ganesan et al. (2022)found that the DMN was less activated during meditation than control conditions in only 60% of the studies reviewed, with the controls including rest, intentional instructions to mind wander, and other functional tasks. A final concern is that reverse inferences from brain regions or networks to psychological processes may be implausible (Poldrack, 2006). Task-based fMRI paradigms have been foundational in identifying brain function**associated**with meditation. However, to build a mechanistic account, researchers need to manipulate brain function (Kvamme et al., 2022), and neurofeedback affords one opportunity to manipulate brain functions directly implicated in mindfulness meditation.

Neurofeedback originated in the 1960s for EEG (Kamiya, 2011), and early 2000s for fMRI (e.g.,deCharms et al., 2004;Yoo & Jolesz, 2002). Similar to biofeedback, it consists of relaying brain data (i.e., the target measure) to a participant while they perform a task. The participant may be given instructions to modulate the target by any number of strategies, or they may be given a specific strategy and told that correct application will be indicated by changes in their brain data. This neurofeedback condition may be compared with control conditions, wherein participants are presented with data from other brain regions not affected by the strategy (“alternative ROI control”), or from other participants (“yoked” sham). Given well-designed controls (Sorger et al., 2019), neurofeedback can provide more substantive evidence that a brain region or network is involved in a process (Kvamme et al., 2022;Sitaram et al., 2017). Specifically, neurofeedback could elucidate the causal links between meditation and brain function, excluding possible spurious associations—but it does not provide evidence that a brain region or network**causes**meditative states, which requires direct brain stimulation (Kvamme et al., 2022;Lord et al., 2024). In addition to providing mechanistic insights into mental processes, neurofeedback allows participants to manipulate those processes. Neurofeedback has been used in many different applications, from the regulation of chronic pain (deCharms et al., 2005) to attentional training (typically involving prefrontal regions) (DeBettencourt et al., 2015;Wang & Hsieh, 2013) to stress reduction (typically involving the amygdala) (Hellrung et al., 2018;Nicholson et al., 2017;Young et al., 2017). It is often considered to “enhance” learning, leading to improved outcomes (Haugg et al., 2021;Kadosh & Staunton, 2019). Researchers often conduct a single session of neurofeedback and then evaluate behavioral outcomes days, weeks, or months later (Pamplona et al., 2023;Ros et al., 2013); though several studies have leveraged repeated sessions (Dekker et al., 2014;Mehler et al., 2018). Overall, there is promising evidence for the clinical mental health benefits of EEG and fMRI neurofeedback (Roy et al., 2020;Trambaiolli et al., 2021;Van Doren et al., 2019; c.f.Thibault et al., 2018). Thus researchers have proposed that the clinical benefits of mindfulness (as well as cognitive benefits) could be enhanced or facilitated by neurofeedback (Brandmeyer & Delorme, 2013;Brandmeyer & Reggente, 2023).

In these proposals, researchers suggest neurofeedback may operate as a “technological mirror” and provide insight into the mind (Brandmeyer & Delorme, 2013;Brandmeyer & Reggente, 2023). It is often the case that beginning meditators experience significant mind-wandering during practice, and mindfulness meditation involves slowly becoming aware of these episodes of mind-wandering and learning to re-orient your attention back to the object of meditation. Perhaps neurofeedback could facilitate this gradual learning process, through operant conditioning (associating behavior with rewards). This is proposed to involve explicit strategies or conscious awareness (improved “meta-awareness”,Brandmeyer & Reggente, 2023), contrasting with evidence of implicit learning of brain modulation in other neurofeedback domains (Sitaram et al., 2017). Facilitated mindfulness may transfer to contexts without feedback (Krause et al., 2024), just as mindfulness practice is empirically demonstrated to lead to improvements in day-to-day mindfulness (Kiken et al., 2015;Quaglia et al., 2016). More generally, neurofeedback may help participants learn dynamic self-regulatory strategies for their day-to-day life and lead to improved mental health (Krause et al., 2024).

Starting in the early 2010s, neurofeedback concurrent with mindfulness meditation has been gaining popularity, and it is often referred to as mindfulness-based neurofeedback (mbNF). The purpose of this paper is to systematically review the literature and thus answer two main questions. First, can participants learn to modulate brain targets through mindfulness meditation practice, providing evidence of their involvement in meditation? Second, what are the behavioral and brain outcomes of mbNF, and do they relate to enhanced learning of mindfulness? We specifically examine mindfulness meditation defined as attentional contemplative practices involving the purposeful redirection of attention to the present moment (Dahl et al., 2015). By reviewing the literature, there also are opportunities to discuss methodological limitations. The CRED-NF checklist (Ros et al., 2020) could be a crucial initial step toward standardizing current methodological and outcome reporting practices. The CRED-NF checklist includes preregistration, sample size justification, control group, double-blinding, whether or not participants used a strategy, artifact removal, feedback specification, regulation success (target engagement), brain and behavioral outcomes, and more. We evaluate the quality of studies herein based on the CRED-NF checklist. The present review only examines controlled laboratory-based EEG and fMRI studies (consumer-grade EEG studies are not reviewed, seeSection 2).

## 2 Methods

PRISMA guidelines were followed in this review (Supplementary 2) (Page et al., 2021).

### 2.1 Inclusion and exclusion criteria

Studies that employed EEG or fMRI neurofeedback concurrently with mindfulness meditation were included. Specifically, studies were selected that claimed to employ mindfulness meditation, and we then evaluated whether the meditation met our definition of mindfulness. For the purposes of this review, mindfulness meditation is defined as meditation practice with the aim of cultivating non-judgmental attention to the present moment, including both focused attention (FA) and open monitoring (OM) practices (Lutz et al., 2008). FA and OM are distinct practices, but both are taught in mindfulness interventions such as MBSR (Santorelli, 2014) and involve purposeful redirection of attention to the present moment (Britton et al., 2018;Dahl et al., 2015). Other meditations (e.g., transcendental, compassion) were not included, as they involve distinct psychological and neural processes (Fox et al., 2016;Garrison et al., 2015;Van Dam et al., 2018). This step was applied to reduce heterogeneity in meditation designs (Sparby & Sacchet, 2022). Exclusion criteria included lack of EEG or fMRI neurofeedback, lack of mindfulness meditation, lack of concurrent neurofeedback and mindfulness meditation, and non-empirical status (e.g., reviews). Studies with consumer-grade EEG devices were considered beyond the scope of this review, as consumer-grade devices do not release information on the ways they calculate and deliver feedback, preventing insight into brain mechanisms. In addition, there are concerns that consumer-grade devices use brain measurements that may be heavily contaminated by non-neural signals (Wexler & Thibault, 2019).

### 2.2 Systematic search

A search of PubMed, Web of Science, PsycInfo, and Scopus was completed on November 11, 2023. Databases were identified based on previous mindfulness systematic reviews and meta-analyses (Goldberg et al., 2018;Treves et al., 2019). Search terms were “(mindfulness OR meditation) AND (neurofeedback OR neural feedback OR neuro feedback)”. We additionally searched reference sections of included papers.

### 2.3 Study selection

All studies were first screened for duplicate publications. Next, all abstracts were screened, including studies based on two main criteria: full report of an empirical study (examples of excluded articles were review papers, protocol papers, book chapters, and conference abstracts) and content relevance (based on above stated inclusion/exclusion criteria). Then remaining studies were screened by reviewingSection 2and full paper to further evaluate the presence of inclusion criteria. Determination of inclusion was established in cases of disagreement by consulting with the first author.

### 2.4 Coding

Records were grouped according to neuroimaging technique (i.e., EEG or fMRI). Two reviewers (KDG & EW) independently evaluated each EEG study and its characteristics, and two reviewers (INT & ZB) independently evaluated each fMRI study and its characteristics. The studies were coded for sample, targets, neurofeedback details, control conditions, target engagement, neural outcomes, and behavioral outcomes (Tables 1and2). Target engagement was defined as “whether or not the neurofeedback target was modulated,” whereas neural outcomes are changes in other neural measures not targeted in the study neurofeedback protocol. Behavioral outcomes may consist of outcomes such as state mindfulness reported after the scan, or more distal but related outcomes (e.g., cognitive performance on a separate task).

**Table 1.** Summary of studies of fMRI-based neurofeedback with concurrent mindfulness practice.

Author/date	Sample	Mindfulness meditation	Control condition	Neural target	Feedback presentation	fMRI session details	Target engagement	Neural outcomes	Behavioral outcomes
Garrison, Scheinost, et al., 2013	Exp1: 9 meditators (variety of traditions, M = 9.5 yrs, 8,803 hrs), 11 non-meditators Exp2: 10 meditators (variety of traditions, M = 18.4 yrs, 10,567 hrs)	Focused attention/breath practice	None ^a^	PCC activation	Bar graph: blue upward bar for activation increases, red downward bar for activation decrease. Full graph with past feedback shown	Exp1: 3-min NF scan. Exp2: three 1-min NF scans	Exp 1: More negative PCC activations during NF in meditators compared with controls. Exp 2: significant deactivation of PCC compared with self-reference	Not reported	Effortless awareness was associated with decreased PCC activity
Garrison, Santoyo, et al., 2013	Same sample as Exp2 from Garrison, Scheinost et al., 2013 , 10 meditators	“	Within-subject no feedback	“	“	Six 1-min feedback scans	Not reported	“	Qualitative report: PCC deactivation was associated with experience of focused attention and effortless awareness, PCC activation was associated with opposite
Kim et al., 2019	60 adults ^b^	Focused attention/ breath practice	Yoked sham group	Individually localized DMN, CEN, and SN. Mediation slope excluding CEN from DMN and SN relationship	Thermometer where higher bar reflects higher mediation slope. Calculated using windowed brain activity from 50 sec prior	Two 5-min NF runs, one transfer fMRI run	Mediation effect not significantly larger in experimental group. However, correlation between mediation effect and mindfulness/target performance feedback (TPF, self-report) only in experimental group	Activations in DMN negatively correlated with mindfulness/TPF in experimental group	No group X time effects on mood, state mindfulness, TPF, or stress. No reported changes in cognitive tasks
Bauer et al., 2020	11 participants with schizophrenia or schizoaffective disorder	Open awareness (noting)	Alternative ROI control: from somatomotor cortex during finger tapping in same participants (7 completed)	Individually localized CEN and DMN networks. Increase CEN relative to DMN (PDA)	A moving ball. The ball moves relative to the difference between CEN and DMN. If CEN > DMN, ball moves up. If DMN > CEN, ball moves down. Activations from 30 sec prior	Two no-feedback transfer runs (2.5-min each), four feedback scans (2.5-min each)	Participants showed significant CEN > DMN (more than chance) on average during NF. Unclear whether control condition also engaged target	Decreased DMN connectivity (mPFC-PCC), decreased CEN-DMN connectivity (dlPFC-mPFC) from pre- to post-resting state, not present in control condition	AHs decreased 1-wk after, returning to baseline after >12-wks. AHs were not affected by control neurofeedback task
Pamplona et al., 2020	30 adults	Focused attention ^c^	No-training control group	SAN activation (composite of DAN and FPN) minus subject-specific DMN (core hubs and angular gyrus). Compared with baseline blocks	Intermittent feedback with thermometer (red is high SAN-DMN, blue is low SAN-DMN) every 40 sec (with monetary rewards at end of run)	Two NF runs (6-min each), two transfer runs	Decreased DMN activations over training (specifically mPFC and PCC) and in post-transfer runs (compared with pre-transfer). Increased attentional network (specifically mid-cingulate and pre-SMA) activations during training and in transfer runs (specifically IPS)	Not reported	Control group improved more on multiple attention tests, but NF group improved in RTs for vigilance test, specifically during early trials. No changes in attentiveness and stress. No relationship between changes in self-report/behavior changes and target engagement
Pamplona et al., 2023	Same sample as Pamplona et al. (2020) , 15 adults	"	None	"	"	"	Transfer at 2 months: DMN deactivation present (PCC/mPFC), not present in SAN	Transfer at 2 months: DMN–visual area correlations increased and maintained at follow-up, related to degree of psychomotor vigilance changes	No behavioral effects persist at 2 months
Kirlic et al., 2022	34 adolescents (ages 13–17 years)	Focused attention/ breath practice	None	PCC activation	Bar graph: blue upward bar for activation increases (“focused attention”), red downward bar for activation decreases (“mind-wandering”). Tasked to match blue bar with green target bar	Three 7-min NF runs, two transfer fMRI runs (OBS and TRS)	PCC deactivation during NF, consistent when compared with rest or self-referential processing. Not observed during post-transfer. Widespread deactivations in other regions. Limited evidence of correlations between PCC activation and self-reports (e.g. mindfulness)—does not survive MC	Not reported	No changes in PSS or negative affect. State mindfulness increase maintained at 1 wk
Yu et al., 2022	Same sample as Kirlic, 37 adolescents (ages 13–17 years)	"	"	"	"	"	"	Posterior insula activations decrease. Anterior insula activations increase. No transfer effects	Self-report state mindfulness increased. No change for mind-wandering
Zhang et al., 2023	9 adolescents (ages 17–19 years) with lifetime history of major depressive disorder/anxiety disorders	Open awareness (noting)	None	Individually localized CEN and DMN networks. Increase CEN relative to DMN (PDA)	A moving ball. The ball moves relative to the difference between CEN and DMN. If CEN > DMN, ball moves up. If DMN > CEN, ball moves down. Activations from 30 sec prior	Five 2.5-min NF sessions. No transfer	More overall time in CEN > DMN state. Marginally lower DMN activation	sgACC-DMN (mPFC/PCC) connectivity decreased. Target performance (PDA) correlated with decrease (only in last NF block)	State mindfulness increased, correlated with target performance and connectivity decrease

a Feedback from parietal cortex only used during monitoring phase.

b All male participants.

c Participants allowed to use any strategy that works for them.

*Note*. AH = auditory hallucinations; CEN = central executive network; DAN = dorsal attention network; dlPFC = dorsolateral prefrontal cortex. DMN = default-mode network; FPN = frontoparietal network; Hrs = hours. M = mean; MC = multiple comparisons; Mdn = median; Min = minute; mPFC = medial prefrontal cortex; OBS = observe runs; PACE = prospective acquisition correction; PCC = posterior cingulate cortex; PDA = positive diametric activity; PSS = perceived stress scale; RT = reaction time; SAN = sustained attention network; SD = standard deviation; Sec = second; SN = salience network; STG = superior temporal gyrus; TPF = task–performance feedback; TRS = transfer runs; Wk = week; Yr = year.

**Table 2.** Summary of studies of EEG-based neurofeedback with concurrent mindfulness practice.

Author/date	Sample	Mindfulness meditation	Control condition	Neural target	Feedback presentation	EEG session details	Target engagement	Neural outcomes	Behavioral outcomes
Hinterberger & Fürnrohr, 2016	26 meditators (M 8.2 yrs practicing), 10 non-meditators	Focused attention and body scan ^a^	Within-person control. Focused attention meditation only, body scan meditation only, and yoked sham	EEG frequency bands (USP, SCP, delta1, delta2, theta, alpha, beta, gamma, wide) amplitude and time from peak-to-peak of a wave cycle	Sensorium (multimodal NF environment using sound and light changes)	Two 20-min sessions on 2 separate days	Compared with the aggregate of non-Sensorium conditions, the aggregate of all Sensorium conditions showed a stronger increase in power in the theta2, alpha1, and alpha2 bands ^b^	Not reported	Subjective feedback ratings show Sensorium was not inferior to meditation alone, and was rated as a more extraordinary experience. The Pseudo-Sensorium was found to be inferior to Sensorium3
Kosunen et al., 2016	43 adults	Focused attention and body scan	Within-person controls. Followed both meditation exercises (1) using a computer screen with no VR headset or NF and (2) using the VR headset with no NF	Increase in power of alpha band and theta band	VR headset. Users begin on a platform. Increases in theta band power correspond to platform levitating, while increases in alpha band power correspond to increases in opacity of energy bubble surrounding user	Two 10-min NF sessions in 1 day	Not reported	Not reported	On a meditation depth questionnaire, the VR+NF condition performed significantly better than the Screen only condition, but not the VR+no NF condition
Salminen et al., 2023	Same sample as Kosunen et al., 2016 , 43 adults	"	"	"	"	"	No significant effect for alpha. Significantly greater frontal theta activation during NF sessions vs. no-NF conditions	Significantly more gamma power during VR vs. computer screen. Significantly more gamma power during NF vs. no-NF. Significantly more gamma power during body scans than focused attention	Higher self-reported sense of presence during NF vs. no-NF. Higher self-reported sense of presence was reported in VR vs. computer screen conditions
van Lutterveld et al., 2017	16 novice meditators, 16 experienced meditators (Mdn 6,164 hrs, minimum 5 yrs experience)	Noting practice (for novices), and effortless awareness (for experienced meditators)	Within-person control. Bidirectional control	Decreased gamma band PCC activity	Bar graph: upward bar for increases in PCC power and downward bar for decreases in PCC power. Full graph with past feedback shown	Three 1.5-min runs of concurrent meditation and NF	Novice meditators were able to decrease PCC power in noting practice runs only. Experienced meditators were able to for all runs. Neither group was able to upregulate PCC (bidirectional control)	Not reported	Both groups associated effortless awareness with decreased PCC activity
Dunham et al., 2018	10 adults	Open awareness	None	BIS value (higher value correlates with higher power in high-frequency bands)	Continuous display of raw EEG and a BIS value (0–100). Participants were told a BIS value more than 94 indicates fast brainwave activity, which might denote stress	Up to 4 days over a 21-day period. Each day has two 12-min blocks	BIS value significantly decreased compared with baseline. For the one participant who completed 4 days, mean BIS score significantly decreased from days 1 to 4	Not reported	For the one participant who completed 4 days, well-being score increased from days 1 to 4
Dunham et al., 2019	57 adults	Open awareness	None	“	“	4 days over a 21-day period. Each day has two 12-min blocks	Mean BIS and minimum BIS lower than baseline BIS for each of 4 training days, but no significant change in values across days	Not reported	Well-being scores significantly increased from days 1 to 4, and were significantly higher than the single time point comparison sample
Prestel et al., 2019	6 meditators (Mdn 70 hrs experience)	Focused attention and open monitoring ^c^	Within-person control. Final session was meditation only with no NF	Increase frontal midline theta (FMT)	Grayscale sphere, increases in FMT power correspond to sphere becoming larger, decreases in FMT power correspond to sphere becoming smaller	Eight sessions over 2 weeks. Each session has five 5-min training blocks	Number of sessions was significantly positively associated with greater FMT power. However, this was mostly due to two subjects who had a strong significant positive correlation, while the other four subjects had non-significant effects. Mixed results for control condition	Not reported	In post-session interviews, some participants reported negative experiences with NF (e.g. distraction, pressure to perform). Subjective appraisal of performance did not always align with one’s FMT power values
Brandmeyer & Delorme, 2020	24 adults	Focused attention/breath practice	Between-person control. Yoked sham NF (from gender-matched pair in the experimental group)	Increase frontal midline theta (FMT)	Colored square, color was updated 4X per sec, with a gradient from black (low FMT amplitude) to light blue (high FMT amplitude)	Eight sessions over 2 weeks. Each session has six 5-min training blocks	NF group had significant increase in FMT activity across sessions. No significant differences in FMT across sessions among sham control group	Significant increase in gamma power in frontal midline and left temporal parietal areas during N-2 back task pre- to post-NF for NF group only. No significant differences in EEG activity for SART or local–global task (attention)	Faster reaction times post-NF on correct trials during the N-2 back working memory task for NF group only. No significant results for SART or local–global task (attention)
Chen et al., 2021	34 meditation-naive participants (17 with anxiety disorder; 17 healthy)	Mindfulness recording therapy ^d^	None	Alpha band power of right and left frontal lobes	Bar graph with two bars representing alpha power on the left (colored red) and right (colored green) sides of the frontal lobe	One 8-min session	Not reported	Significant increase in alpha, gamma, and theta power pre–post mindfulness NF for both groups. An ANOVA revealed a significant main effect of condition (anxiety vs. healthy), condition × brain region, condition x hemisphere, and condition x region x hemisphere	Not reported

a Mindfulness instructions for control conditions were different than instructions for Sensorium conditions, which were more general.

b Two of the three Sensorium conditions utilized neurofeedback (Sensorium 1 condition did not).

c Participants allowed to use any strategy that works for them.

d Details of mindfulness task were not further specified.

*Note.*BIS = Bispectral Index™; EEG = electroencephalography; FMT = frontal midline theta; Hrs = hours; M = mean; Mdn = median; Min = minutes; NF = neurofeedback; PCC = posterior cingulate cortex; SART = sustained attention to response task; SCP = slow cortical potentials; Sec = seconds; USP = ultra-slow potentials; VR = virtual reality; wide = 1–40 Hz. Yrs = years.

### 2.5 Bias and quality coding

No automation tools were used. Papers were coded independently to limit reviewer bias. Risk of bias in the studies was not quantified given the limited number of RCTs. Instead, we coded studies based on the CRED-NF checklist (Ros et al., 2020), reporting whether recommended items were present in the studies (Supplementary Table S1).

## 3 Results

### 3.1 Search results

A PRISMA flow diagram is shown inFigure 3. The search yielded 676 records across 4 databases. After removing duplicates and excluding based on title and abstract, full texts were reviewed for the remaining 114 studies. The final sample included 18 studies with 15 independent samples representing 419 participants.

**Fig. 3.**
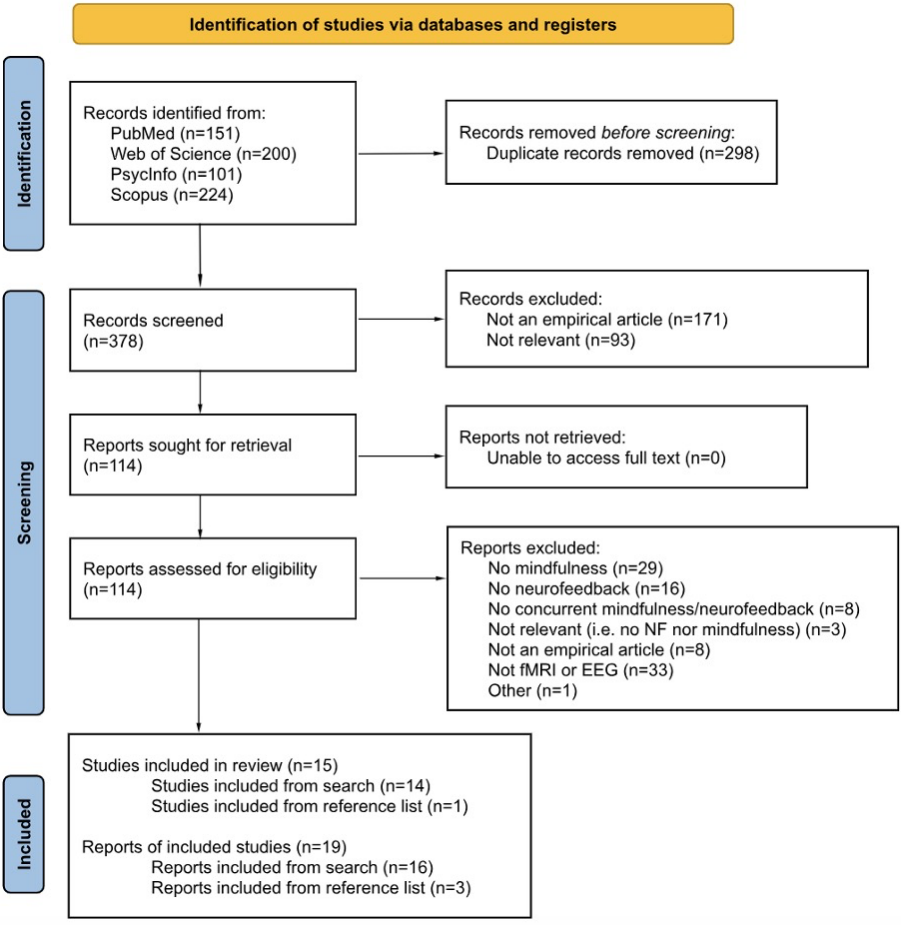
PRISMA flow diagram depicting number of identified and evaluated articles for concurrent mindfulness and fMRI or EEG neurofeedback procedures.*Note*. Studies refer to unique samples, while reports refer to publications on said samples. Our review identified four samples which corresponded to more than one published report, as indicated in this flowchart and in the study summary tables (Tables 1and2).

### 3.2 fMRI studies

#### 3.2.1 Summary

We identified nine reports of fMRI neurofeedback including seven unique samples (Table 1). In total, there were 177 unique participants, and most samples were at or below 30 participants (Supplementary Fig. S1). Studies employed focused attention and open monitoring meditations. The first study published wasGarrison, Scheinost, et al. (2013), a breath-focused attention study, which was also the only study to involve experienced meditators.Garrison, Santoyo, et al. (2013)examined qualitative reports from meditators who were asked to explore correspondence between brain signals and meditation experiences. A typical open monitoring protocol asked participants to label thoughts and feelings as they came up (Bauer et al., 2020). Only one study reported participants’ actual mental strategies during neurofeedback regulation (Pamplona et al., 2020), and found a variety of strategies employed, some of which were not typical mindfulness. Control conditions encapsulated yoked sham, alternate ROI, and mindfulness meditation without feedback, but many studies did not include controls (Supplementary Fig. S2). Only two studies examined between-subjects controls (Supplementary Fig. S3) (Kim et al., 2019;Pamplona et al., 2020), and transfer runs (Box 1) were inconsistently used across studies. Three reports examined adolescents (Kirlic et al., 2022;Yu et al., 2022;Zhang et al., 2023). Qualitative assessments of the mbNF experience are found inSupplementary Table S4.

Box 1. Neurofeedback Terms

**Bidirectional control:**Testing whether participants may modulate a neurofeedback target in both directions. For example, decreasing the DMN by meditating, and increasing the DMN by ruminating.

**Calibration:**A preceding block of non-neurofeedback data used for the neurofeedback target estimates, typically eyes-open rest.

**Control, Alternate ROI:**Typically, feedback is given from a region or network that is not related to the task.

**Control, Yoked Sham:**Feedback is presented to a control participant from an experimental participant. This feedback is controlled for in terms of perceived reward but not contingent on a control participant’s performance.

**Functional/individual localization:**Determining a brain area or network based on data from the participant. An example is conducting resting-state fMRI before the neurofeedback task, which can be used to extract intrinsic networks that are correlated at rest.

**Intermittent vs continuous:**Intermittent, or delayed, feedback is feedback presented after regular intervals, not concurrently with task. Continuous, or real-time, feedback is feedback presented throughout the task (e.g. every second), may involve different attentional demands (Hellrung et al., 2018).

**Offline artifact correction:**Estimates of motion or physiology are corrected for or tested for in post-processing.

**Online artifact correction:**Estimates of motion or physiology are included in real-time models (e.g. GLMs), so feedback is not presented based on those artifacts.

**Target:**The brain measure relayed to participants.

**Target engagement:**A test of whether participants successfully learned to modulate the target brain measure, may consist of examining overall levels of target, change in target, or target performance in transfer runs.

**Transfer run:**A neuroimaging run where participants perform the neurofeedback task without any feedback presented. Transfer tasks after feedback can be used to assess whether learning has occurred.

#### 3.2.2 fMRI targets

**Fig. 4.**
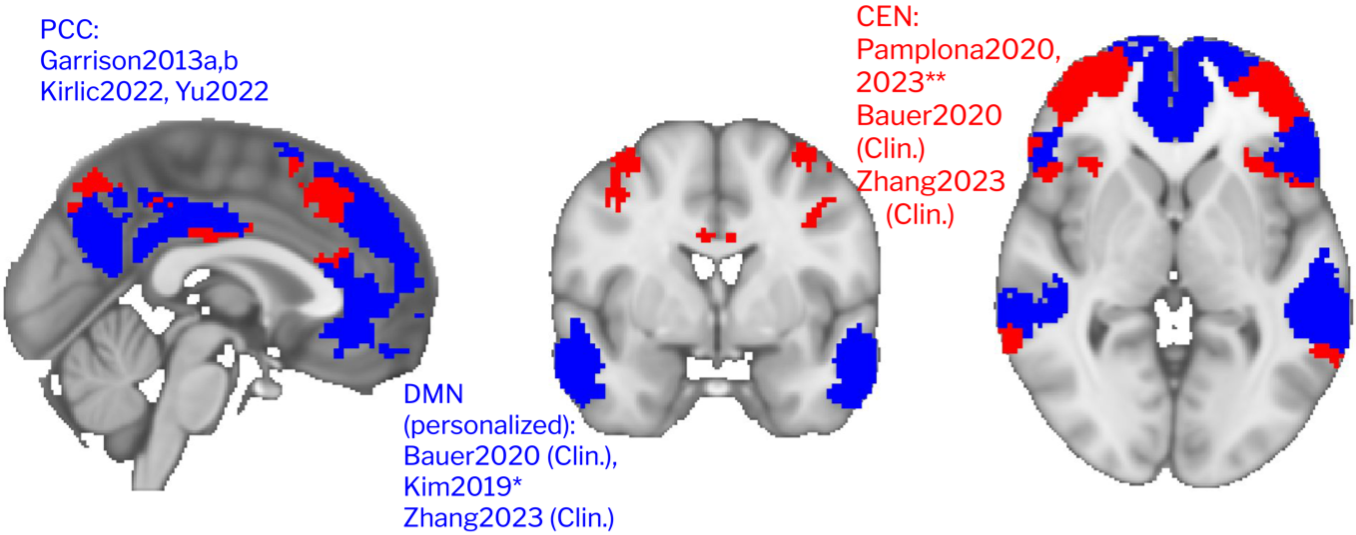
fMRI targets for neurofeedback. In blue, default-mode network (DMN) regions. In red, the central executive network (CEN). Networks are taken from the Yeo atlas (Thomas Yeo et al., 2011). *Kim used DMN-SN slope, **Pamplona used sustained attention network.

The majority of studies employed activation-based, default-mode network targets (Fig. 4). There was some variety in the specification of the DMN. Two studies (four reports) targeted PCC activity (Garrison, Santoyo, et al., 2013;Garrison, Scheinost, et al., 2013;Kirlic et al., 2022;Yu et al., 2022). Multiple studies used individualized networks generated from independent component analysis of resting-state scans (Bauer et al., 2020;Kim et al., 2019;Pamplona et al., 2020,^2023^). These studies combined network activations from not only the DMN but also from attentional networks such as the dorsal attention network (Pamplona et al., 2020,^2023^), the salience network (Kim et al., 2019), and the CEN (Bauer et al., 2020).Kim et al. (2019)was the only study which used a connectivity-like target, and they examined the direct effect from DMN-SN, excluding the influence of the CEN (as the CEN was proposed to be involved with the visual feedback monitoring and not the meditation). Target measures did not appear to depend on whether participants performed focused attention versus open monitoring.

There were different approaches to computing the target measure. Multiple studies used baseline periods in the same fMRI scan to scale and baseline the neurofeedback target measure (e.g., 30 sec of rest;Bauer et al., 2020;Zhang et al., 2023).Garrison, Santoyo, et al. (2013)andGarrison, Scheinost, et al. (2013)used a self-reference task. Some studies used online motion artifact correction, and two studies additionally conducted online correction for physiological signals such as breathing and heart rate (Kirlic et al., 2022;Yu et al., 2022). No differences were observed between neurofeedback and rest in terms of motion or physiological signals.

Feedback was displayed visually to participants during fMRI in all cases. Some studies used delayed feedback, for example, continuous feedback but with a time lag (Bauer et al., 2020;Kim et al., 2019), and one study used intermittent feedback (Pamplona et al., 2020). One study displayed a continuously updating graph containing past values of the measure (Garrison, Scheinost, et al., 2013). Negative and positive feedback were shown. One study incorporated rewards for target engagement (Pamplona et al., 2020).

#### 3.2.3 fMRI target engagement

All studies evaluated target engagement.Garrison, Scheinost, et al. (2013)found that meditators showed more negative PCC (the target) activation than controls. Two studies examined the amount of time spent in a “correct” brain state (CEN > DMN), and found above chance engagement for the group (Bauer et al., 2020;Zhang et al., 2023). Another study examined change over neurofeedback blocks in activation, and found significant decreases in the DMN (Pamplona et al., 2020), but no increases in their individually defined attentional networks. Transfer runs can also be used to assess whether target engagement or learning has taken place.Pamplona et al. (2020)observed decreases in the DMN compared with pre-training, which persisted at 2 months follow-up (Pamplona et al., 2023).Kirlic et al. (2022)observed decreased PCC activation during neurofeedback compared with control tasks, but not in the post-transfer run. Although there seems to be consistent evidence of DMN deactivation during neurofeedback, the only study to use a sham control and a large (n = 60) sample did not find evidence for target engagement (mediation slope between the DMN-SN) (Kim et al., 2019). This and the transfer results from Kirlic and colleagues make it unclear whether participants have learned to modulate their brain networks.

#### 3.2.4 Neural outcomes

Many studies examined the possibility of neural changes due to neurofeedback. Some reports focused on DMN-based connectivity assessed during resting-state fMRI before and after neurofeedback. There are indications of reduced within-DMN connectivity (e.g., between the MPFC and PCC), as well as more negative correlations between DMN and CEN (Bauer et al., 2020). One study found increased DMN–visual area connectivity even at a 2-month follow-up assessment (Pamplona et al., 2023).Zhang et al. (2023)found reduced DMN-sgACC connectivity, which correlated with target engagement and state mindfulness in their small sample.

Researchers also examined activations in non-target and target brain regions.Yu et al. (2022)found that neurofeedback increased anterior insula activations, and decreased posterior insula activations, without any transfer effects. This could reflect changes in interoceptive processing during mbNF.Kim et al. (2019)found that activations in the DMN negatively correlated with state mindfulness, but only in the experimental group and not the sham feedback group.

#### 3.2.5 State mindfulness

To assess whether participants are learning from neurofeedback, studies also tested whether they experienced increases in state (or momentary) mindfulness, as reported after the scans. State mindfulness assessments typically involved questions about present-focused awareness of the mind and body (Tanay & Bernstein, 2013).Kim et al. (2019)found no mbNF versus control group effects on state mindfulness or self-report target efficacy. However, two uncontrolled studies found increases in state mindfulness after neurofeedback (Kirlic et al., 2022;Zhang et al., 2023).

#### 3.2.6 Behavioral outcomes

A central motivation for many of the studies was the possibility of beneficial behavioral or self-reported outcomes.Pamplona et al. (2020)observed improvements in reaction time on a vigilance test, but this was not maintained at the 2-month follow-up (Pamplona et al., 2023). They also did not observe a correlation between target engagement and vigilance reaction time.Kirlic et al. (2022)did not observe any changes in perceived stress or negative affect after neurofeedback.Bauer et al. (2020)observed decreases in auditory hallucinations that were not present after a control neurofeedback task. Overall, there is limited evidence for mindfulness-based fMRI neurofeedback benefits as yet.

#### 3.2.7 Clinical applications

Two studies conducted neurofeedback with small clinical samples (Bauer et al., 2020;Zhang et al., 2023).Bauer et al. (2020)examined neurofeedback in 10 individuals with schizophrenia, and found decreases in auditory hallucinations—although these changes were not sustained at 12 weeks. Zhang et al. examined neurofeedback in nine adolescents with affective disorder history, and found decreases in sgACC-DMN connectivity which is heavily implicated in adolescent depression (Chai et al., 2016), though symptom changes were not assessed. These studies can be considered pilots—focused mostly on establishing feasibility of the neurofeedback protocols in clinical samples.

#### 3.2.8 Quality (CRED-NF)

In general, the control conditions in fMRI studies (within and across subjects) were lacking, with only one study involving adequate controls and reporting. Reporting of feedback specifications, target engagement (in the feedback condition), data processing methods, etc. was present across the vast majority of studies. Few studies conducted pre-registration, power analyses, or made their data/code open access. A full table can be found inSupplementary Table S2.

### 3.3 EEG studies

#### 3.3.1 Summary

Nine reports of EEG neurofeedback during mindfulness meditation were identified, corresponding to eight unique samples (Supplementary Fig. S1). In total, there were 242 unique participants, and all samples were adults. Multiple samples looked at meditators, or compared meditators with non-meditators (Hinterberger & Fürnrohr, 2016;Prestel et al., 2019;van Lutterveld et al., 2017). Only one sample included a clinical population (anxiety disorders;Chen et al., 2021) and only two samples did not include a control condition (Chen et al., 2021;Dunham et al., 2018;Dunham et al., 2019). All control conditions were within subject with the exception ofBrandmeyer and Delorme (2020)(Supplementary Fig. S3). However, the control conditions varied; most were compared with some form of meditation without neurofeedback and others included yoked shams (Supplementary Fig. S2) (Brandmeyer & Delorme, 2020;Hinterberger & Fürnrohr, 2016). The most common types of meditation were focused attention, body scan, and open monitoring. The terminology for the type of meditation was not always consistent, and we used specific reporting from studies to classify meditation types. That said, reporting on specific mindfulness instructions was not always clear (Chen et al., 2021) and participants were sometimes allowed to use various strategies (Prestel et al., 2019). It is also important to note that even within a single study, the instructions of the control condition mindfulness did not always match the instructions of the active NF session (Hinterberger & Fürnrohr, 2016). Qualitative assessments of the mbNF experience for EEG are given inSupplementary Table S5.

#### 3.3.2 EEG targets

**Fig. 5.**
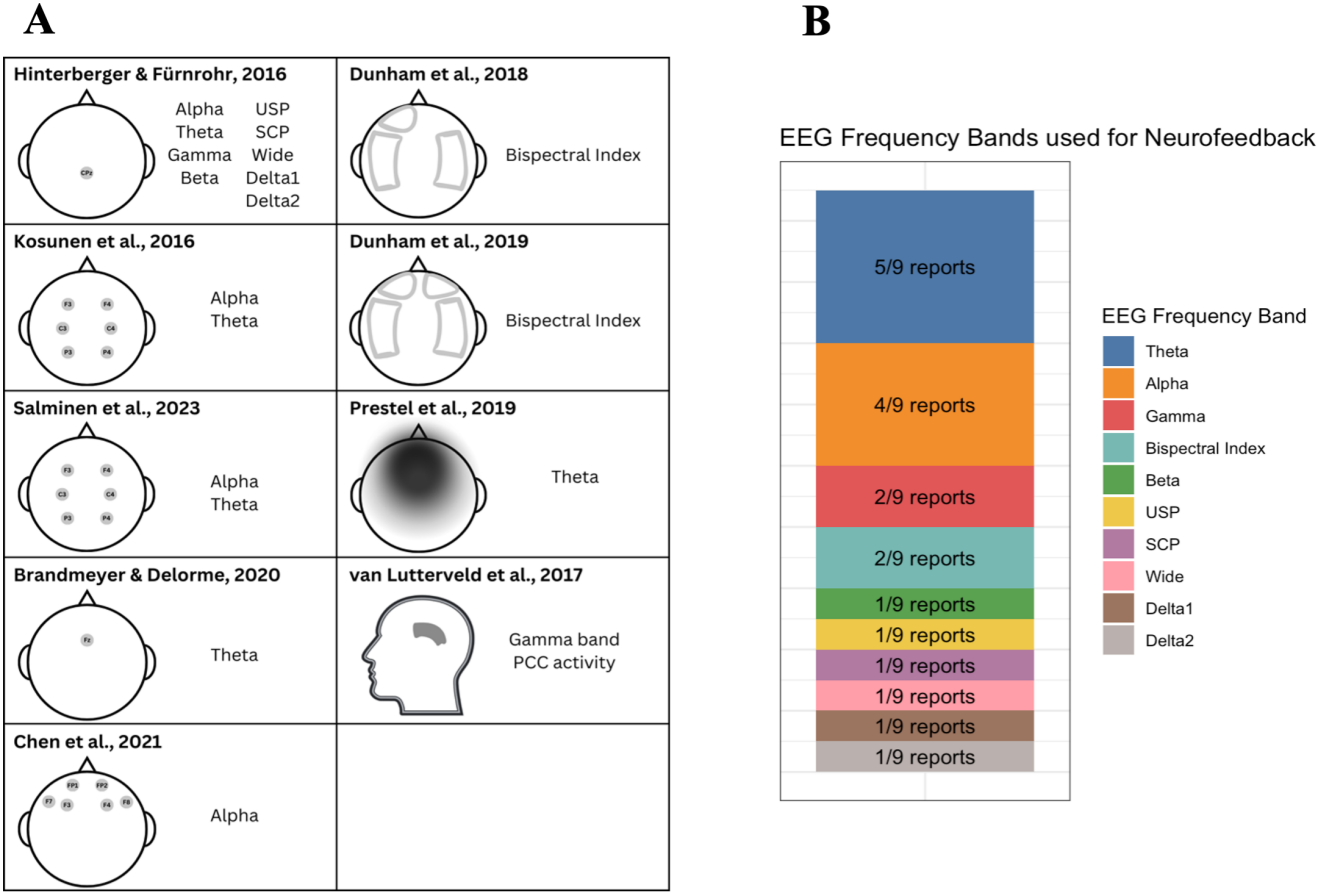
This figure displays the variety in methods of calculating EEG-neurofeedback. In (A), the spatial layouts of the neurofeedback targets are displayed.Hinterberger and Fürnrohr (2016)calculated alpha, theta, beta, gamma, USP, SCP, wide, delta1, and delta2 from CPz.Kosunen et al. (2016)calculated theta and alpha using an average from F3, F4, C3, C4, P3, and P4.Salminen et al. (2023)calculated theta power using an average F3 and F4, while alpha power was calculated as an average of F3, F4, C3, C4, P3, and P4.Brandmeyer and Delorme (2020)used Fz to calculate frontal midline theta.Chen et al. (2021)used FP1, F3, F7 and FP2, F4, F8 to calculate alpha of the left and right frontal lobes, respectively.Dunham et al. (2018)placed the BIS sensor on the left forehead and the temporal fossa.Dunham et al. (2019)placed the BIS sensor on the left or right forehead and the temporal fossa.Prestel et al. (2019)used independent component analysis (ICA) with a 32-channel setup to calculate frontal midline theta.van Lutterveld et al. (2017)used source estimation with a 128-channel setup to determine gamma band PCC activity. In (B), the frequency bands used by each report are displayed. Theta was used the most by five reports, closely followed by alpha, which was used in four reports.

Almost all studies used changes in frequency band power as their neural target (Fig. 5); the most common was alpha and theta, though some studies used gamma (van Lutterveld et al., 2017) or Bispectral Index^TM^(BIS) value, which is an EEG technique most commonly used to measure depth of consciousness for patients under general anesthesia (Dunham et al., 2018,^2019^). Multiple studies focused on more than one frequency band (Hinterberger & Fürnrohr, 2016;Kosunen et al., 2016;Salminen et al., 2023). Some studies focused on whole brain frequency band power, while others looked at frontal midline sites (Brandmeyer & Delorme, 2020;Prestel et al., 2019) or source localized areas such as the PCC (van Lutterveld et al., 2017). The density of EEG ranged from high-density 128-channel (van Lutterveld et al., 2017) to extremely low-density Bispectral Index, which generally has 2–4 channels though the exact number of channels was not reported in this case (Dunham et al., 2018,^2019^). Notably, the way the target was calculated varied, even within a sample. For example,Salminen et al. (2023)calculated theta power from an average of two electrodes (F3 and F4) and alpha power from an average of all electrodes (F3, F4, C3, C4, P3, and P4). Other studies used an independent component analysis (ICA) to calculate the target (Chen et al., 2021;Prestel et al., 2019). Interestingly, the only two samples that had the same neural target (frontal midline theta) calculated the feedback differently, withBrandmeyer and Delorme (2020)using the signal from a single frontal electrode (Fz) whilePrestel et al. (2019)used an ICA to determine frontal midline theta. Almost all feedback was displayed visually, most commonly on some sort of screen, though virtual reality was also used (Kosunen et al., 2016;Salminen et al., 2023). One study used both sounds and light changes as their feedback presentation (Hinterberger & Fürnrohr, 2016). Positive and negative feedback were shown for all studies.

#### 3.3.3 EEG target engagement

Not all papers reported on target engagement (Chen et al., 2021;Kosunen et al., 2016). For those that did, the way the target engagement was reported differed. For example, some studies looked at activation compared with baseline (Dunham et al., 2018,^2019^;van Lutterveld et al., 2017), compared with no NF conditions (Salminen et al., 2023), or across sessions using different linear models (Brandmeyer & Delorme, 2020;Prestel et al., 2019). The difference in reporting and measures used makes synthesizing results difficult, as well as the fact that support for target engagement was mixed. The strongest support was for an impact on theta (Brandmeyer & Delorme, 2020;Hinterberger & Fürnrohr, 2016;Salminen et al., 2023;Prestel et al., 2019), while alpha had both significant and null results (Hinterberger & Fürnrohr, 2016;Salminen et al., 2023). However, it is important to noteHinterberger and Fürnrohr’s (2016)significant results were collapsed across multiple experimental conditions, one of which did not include NF. Others found significant changes in BIS values compared with baseline (Dunham et al., 2018,^2019^), but the significant decrease from days 1 to 4 (Dunham et al., 2018) did not hold up in a larger sample (Dunham et al., 2019). For those who did find significant results across days, the significant effect was in one case driven by two participants (Prestel et al., 2019), and was stronger when certain “non-responders” were excluded (Brandmeyer & Delorme, 2020). It is also important to note the spatial limitations of neural targets using EEG, given that spatial resolution of EEG is limited even when using source localization. For example, the one study that used source localization of the PCC found that over 80% of runs examined had significant correlations between the right lateral occipital cortex and the PCC, though less than 36% of runs showed significant correlation between the left supplementary motor area and the PCC (van Lutterveld et al., 2017). Correlations between 40–57 Hz PCC time series and delta, theta, alpha, and beta were calculated, but never surpassed more than 40% of runs showing significant correlations. The frequency specific to the PCC may be more accurate than the source localization, which may be capturing signals from occipital regions of the brain more broadly.

#### 3.3.4 Neural outcomes

Only four studies out of nine reported on other neural outcomes. All four studies examined EEG bands beyond the bands of the neural target, focusing on (a) other frequency bands during NF and/or control (Salminen et al., 2023;van Lutterveld et al., 2017), (b) frequency bands at rest before and after NF (Chen et al., 2021), and (c) frequency bands in tasks before and after NF (Brandmeyer & Delorme, 2020). The following results discuss frequency bands that were not the target of NF. There is support that alpha, theta, and gamma power significantly increase from pre- to post-mindfulness NF (Chen et al., 2021), as well as support for gamma increases during NF compared with no-NF (Salminen et al., 2023). The delivery of NF (virtual reality (VR) > computer screen) and the type of meditation (body scan > focused attention) can also have an impact on the level of gamma (Salminen et al., 2023). There is mixed support for NF’s effect on cognitive tasks, with no significant changes found for attentional tasks, but a significant increase in gamma power during a working memory task done before and after NF for those that received NF (Brandmeyer & Delorme, 2020).

#### 3.3.5 Behavioral outcomes

All but one study reported on some sort of behavioral outcome. Most focused on how individuals felt doing the mbNF (Hinterberger & Fürnrohr, 2016;Kosunen et al., 2016;Prestel et al., 2019;Salminen et al., 2023), while others looked at changes in well-being (Dunham et al., 2018,^2019^) or even performance on cognitive and attentional tasks (Brandmeyer & Delorme, 2020). When looking at immersive ways to deliver neurofeedback, such as the Sensorium or VR, findings suggest participants find these types of modalities more extraordinary, more engaging, have more positive experiences, and less negative experiences compared with audio/visual guided meditations without VR or Sensorium enhancements (Hinterberger & Fürnrohr, 2016;Kosunen et al., 2016;Salminen et al., 2023). However, this difference is not always due to the addition of neurofeedback, as control conditions with these enhancements with yoked sham or no NF did not always show significant differences (Hinterberger & Fürnrohr, 2016;Kosunen et al., 2016). Accordingly, some participants report that too much focus on the NF can be distracting and lead to poorer performance; however, it is interesting to note that subjective experience of performance did not always align with objective performance, as measured by frontal midline theta (Prestel et al., 2019). Beyond experiences during mbNF, studies have found that well-being scores increase from baseline to completion of all sessions, and are significantly higher at completion than a control group who received no mbNF (Dunham et al., 2018,^2019^). There is also some evidence to suggest NF may help improve performance on memory tasks (NF group compared with sham control had faster reaction times post-NF for correct trials during the N-2 back working memory task) (Brandmeyer & Delorme, 2020). However no significant effects were found for attention tasks.

#### 3.3.6 Clinical applications

Only one study examined EEG neurofeedback in a clinical sample of 17 individuals with anxiety disorders (Chen et al., 2021). Compared with healthy controls, anxious subjects exhibited initial lower power in alpha, theta, and gamma. After NF, anxious subjects significantly increased power across all bands in all brain areas.Chen et al. (2021)suggest that the increase in gamma power indicated a reduction in anxiety symptoms, though they did not report on changes in subjective measures of anxiety. ANOVAs revealed interactions of condition versus brain region/hemisphere, but the direction of differences was not reported. Although the remaining studies reported on non-clinical samples,Dunham et al. (2018,^2019^) examined well-being as a target for mindfulness neurofeedback among physicians/nurses, a group within which stress and emotional exhaustion are common (Dunham et al., 2018). Participants’ subjective well-being was found to improve following the mbNF, suggesting that even in non-clinical samples, NF may be a promising avenue to increase well-being.

#### 3.3.7 Quality (CRED-NF)

There were control conditions in the majority of the EEG studies, but they typically lacked blinding (sham). Reporting of feedback specifications and target engagement was common. Few studies reported artifact correction. Few studies conducted pre-registration, justified their sample sizes, or made their data/code open access. A full table is given inSupplementary Table S3.

## 4 Discussion

Mindfulness meditation consists of purposefully bringing one’s attention back to the present moment, and cultivating an open-minded and non-judgmental attitude (Creswell, 2017). Though mindfulness meditation is increasingly used for promoting mental health, there are many open questions about its neural bases. In this review, we investigate a promising tool for understanding the neural mechanisms of mindfulness, neurofeedback. Neurofeedback consists of relaying neural signals (the target) to the participant and examining whether they can learn to modulate the signals (target engagement). Successful modulation provides evidence that a target brain region is involved in meditation. In addition, given the right targets, neurofeedback may help participants practice correctly and lead to better attention, deeper mindfulness, and positive behavioral outcomes. In this systematic review, we assess whether participants can modulate brain targets (insight into neural mechanisms) and whether participants benefit from the practice (behavioral outcomes). We included studies utilizing mindfulness meditation with concurrent EEG or fMRI feedback (i.e., mindfulness-based neurofeedback [mbNF]).

The search yielded 18 reports, with 15 independent samples. The earliest study was published in 2013, underscoring the nascency of the mbNF field (systematic inquiry of neurofeedback more generally extends back to the early 2000s for fMRIs and the 1960s for EEG). Studies used a wide range of targets across brain areas and frequency bands, and often reported different metrics of target engagement. Neurofeedback duration and number of runs varied (from single 15-min sessions to multiple weeks of training). Sample sizes were generally small, given the resource-intensive nature of neurofeedback. Few studies were RCTs, which are critical for establishing mbNF efficacy and testing mechanisms.

### 4.1 Brain targets

One of the prominent neuroscientific theories of mindfulness posits that successful practice leads to downregulation of the DMN, perhaps most robustly the core hubs of the PCC and mPFC (Ganesan et al., 2022). The DMN has a well-established role in internally generated, self-referential thought (Andrews-Hanna, 2012;Buckner et al., 2008). Mindfulness meditation involves recognizing self-referential thoughts, disengaging from them, and engaging in attention on an object like the breath. Thus, mindfulness may involve downregulating DMN activity. Accordingly, many fMRI studies of mbNF chose to target the DMN. Some studies calculated and displayed anatomically defined PCC activations compared with a control self-reference condition (Garrison, Santoyo, et al., 2013;Garrison, Scheinost, et al., 2013), whereas others used subject-specific, functionally derived maps of the DMN (Bauer et al., 2020;Kim et al., 2019;Pamplona et al., 2020;Zhang et al., 2023). Consistent downregulation of the DMN was found. Neurofeedback studies often included other networks such as the central executive network (CEN) and salience network (SN). There is extensive reason to believe that DMN and other network interactions may be involved in mindfulness meditation, specifically in the switching between external and internal modes of attention (Hasenkamp et al., 2012;Mooneyham et al., 2016;Rahrig et al., 2022). Accordingly, studies relayed the participants’ difference between CEN and DMN (Bauer et al., 2020;Zhang et al., 2023), sustained attention networks and DMN (Pamplona et al., 2020,^2023^), and the slope of the DMN–SN connectivity excluding the CEN (Kim et al., 2019). Participants may only partially modulate multivariate target measures - one study found that the DMN was modulated but not the sustained attention network (Pamplona et al., 2020).

Researchers also examined neuroplastic changes dependent on their DMN-based neurofeedback, finding changes in DMN region connectivity with other brain areas such as the anterior cingulate cortex (Zhang et al., 2023). These changes suggest that neurofeedback may modulate intrinsic features of the DMN, offering a key inroad to mitigate ruminative and depressogenic perseveration tendencies (Zhang et al., 2021).

Mindfulness meditation has been associated with power increases in alpha, theta, and gamma waves during meditation (Chiesa & Serretti, 2010;Lee et al., 2018;Lomas et al., 2015;Stapleton et al., 2020). Alpha and theta power may correspond to shifting attention to internal sensations and thoughts (Lomas et al., 2015), whereas gamma power may reflect wider awareness (Lomas et al., 2015;Lutz et al., 2004;Stapleton et al., 2020). There is considerable evidence that gamma EEG activity can be contaminated by muscle activity (Muthukumaraswamy, 2013;Whitham et al., 2007). However, it is not necessary to disregard gamma power altogether, as long as multiple precautions are taken to remove muscle artifacts and confirm they are not correlated with data (e.g.,van Lutterveld et al., 2017). Accordingly, the nine EEG studies of mbNF selected alpha, theta, and gamma targets. The most consistent evidence was for theta increases, specifically frontal midline theta, which is often an indicator of cognitive control (Cavanagh & Frank, 2014). Results were mixed when probing alpha and gamma power.

The study of EEG and fMRI has often been conducted in isolation, and each has advantages and disadvantages. EEG-neurofeedback is useful for precise temporal modulation as well as cost-effective application, but lacks spatial specificity and may be susceptible to motor artifacts (Muthukumaraswamy, 2013;Whitham et al., 2007). fMRI-neurofeedback is useful for targeting specific brain regions with specificity; however, it is expensive and the underlying signals are slow to change. There have been meaningful efforts to develop EEG measures with spatial specificity. Frontal midline theta may be negatively correlated with DMN activation (Prestel et al., 2018;Scheeringa et al., 2008), while gamma may be positively correlated with DMN activation (Berkovich-Ohana et al., 2012). One neurofeedback study included,Van Lutterveld et al. (2017), specifically targeted activity in the PCC by using source localization. Yet, they did find that occipital cortex activity correlated heavily with localized PCC activity. It may be necessary to conduct EEG-fMRI fusion experiments to develop better measures. In a seminal paper,Keynan et al. (2019)created an EEG target measure of amygdala activity derived from machine learning based on simultaneous EEG-fMRI and showed that participants could modulate the target. The amygdala-EEG neurofeedback led to increases in emotional awareness and regulation and decreases in amygdala activation as measured by fMRI.

### 4.2 Brain target summary and limitations

Extant research has, at times, corroborated neuroscientific theories of mindfulness; however, the majority of research did not include robust control conditions, which results in a lack of specificity. For example, decreases in DMN activation during neurofeedback do not indicate that participants are learning or that DMN deactivation is linked to mindful states. One possibility is that focusing on the display of the feedback itself may lead to DMN decreases. There is substantial evidence that engaging in external tasks leads to decreases in DMN activations (Raichle, 2015;Whitfield-Gabrieli & Ford, 2012), and likely changes in power as well (Fitzgibbon et al., 2004;Khader & Rösler, 2011). Another possibility is that mindfulness meditation leads to decreases in DMN activation, but that this process is implicit and beyond conscious control (thus, neurofeedback would not make a difference). To obviate these concerns, researchers need to employ blinded control conditions or/and transfer tasks. Gold-standard control conditions involve delivering participants feedback that should be unaffected by meditation (e.g., activations from another brain area, from another subject, or reversed activation) (Sorger et al., 2019;Thibault et al., 2016). A weaker control condition is mindfulness-as-usual, which is effective for examining general neurofeedback mechanisms, and neurofeedback benefits, but not target-specific mechanisms (Ros et al., 2020). Transfer tasks involve asking participants to meditate but removing the influence of neurofeedback—and one can examine differences in transfer tasks assessed before and after neurofeedback. Notably, the fMRI studies that employed sham controls or transfer tasks did not find significant differential evidence for target engagement (Kim et al., 2019;Kirlic et al., 2022). EEG studies did not employ transfer tasks, and only one study employed sham (Brandmeyer & Delorme, 2020).Brandmeyer and Delorme (2020)found evidence of increased target engagement of frontal midline theta in mbNF, while the sham group showed no significant changes. Comparisons of EEG-neurofeedback with mindfulness-as-usual also resulted in improved target engagement (Hinterberger & Fürnrohr, 2016;Salminen et al., 2023). In summary, there is not currently evidence from the strongest designs supporting mbNF-specific mechanisms of DMN activation control, while there are some indications of control over frontal midline theta.

### 4.3 State mindfulness

It is critical to identify whether neural feedback can engage the proposed target mechanism, but this is insufficient if it does not yield greater mindfulness and associated mental health benefits. Ideally, target engagement also leads to increases in state mindfulness, or deeper mindfulness during practice. There is only limited evidence in our included studies for increased state mindfulness (Hinterberger & Fürnrohr, 2016;Kim et al., 2019;Kirlic et al., 2022;Zhang et al., 2023; c.f.,Kim et al., 2019;Prestel et al., 2019). One concern is that monitoring of the feedback may cause distraction during meditation. For this reason, some studies provided feedback intermittently after blocks of meditation (Pamplona et al., 2020,^2023^), or allowed practitioners to close their eyes during meditation (van Lutterveld et al, 2017). The studies mostly used visual feedback, which may be distracting. Future research could examine the impact of design choices on state mindfulness during mbNF, including visual/auditory modality and continuous versus intermittent feedback. It may also be useful to collect data throughout the course of mbNF to assess inattention.

### 4.4 Behavioral outcomes

Of note, mbNF has often been proposed to enhance mindfulness acquisition (Brandmeyer & Delorme, 2013). A prime motivation for many of the studies reviewed was the possibility of beneficial outcomes in cognition and affect. An fMRI study observed some improvements in reaction times on a cognitive task beyond a control condition (Pamplona et al., 2020), but it was not maintained at follow-up. An EEG study identified memory improvements but not RT improvements (Brandmeyer & Delorme, 2020). Another fMRI study tested perceived stress and negative affect, and did not observe any improvements (Kirlic et al., 2022), whereas an EEG study identified improvements beyond a waitlist control (Dunham et al., 2018). An fMRI study observed decreases in auditory hallucinations (Bauer et al., 2020), a striking finding with implications for deleterious psychosis symptoms. Interestingly, a neurofeedback study on the same sample that employed mindfulness-related strategies to ignore recorded voices also found decreases in hallucinations (and decreased DMN–auditory cortex connectivity) (Okano et al., 2020). Of course, studies may have measured cognitive and affective outcomes but not reported them (many EEG studies did not report behavioral outcomes). Pre-registration of measures and analyses was scarce. Overall, there is limited existing evidence for mindfulness-based neurofeedback benefits in terms of behavioral or clinical outcomes.

### 4.5 Clinical relevance

Clinical populations may benefit from adaptations of mindfulness instruction. Individuals with histories of trauma may experience traumatic re-experiencing and distress due to meditation (Treleaven, 2018;Zhu et al., 2019). Ruminative individuals with a tendency to engage in repetitive negative thoughts may particularly have trouble learning meditation (Alleva et al., 2014;Crane & Williams, 2010;Hilton et al., 2017). It may be especially helpful for these clinical populations to have scaffolds while they meditate. Mindfulness-based NF may be such a scaffold, providing an engaging external locus of attention plus the same essential components of mindfulness—redirection of attention and non-judgment. Studies on mbNF included here involved clinical participants (Bauer et al., 2020;Zhang et al., 2023) but none involved healthy control groups. Future studies should assess directly whether the benefits of mbNF are more pronounced in clinical groups. Of course, mbNF should not be considered a replacement for more traditional mindfulness training (e.g., with in-person teaching). There is a rich psychotherapeutic literature on developing mindfulness adaptations for clinical groups (e.g., mindfulness-based cognitive therapy;Segal et al., 2004) and acceptance and commitment therapy (Hayes et al., 1999), and mbNF requires more validation before joining these frontline treatments.

### 4.6 Summary

This systematic review of mindfulness meditation concurrent with EEG or fMRI neurofeedback suggests that participants can learn to downregulate the DMN and increase power in the theta band. However, the lack of adequate control conditions limits mechanistic assertions. In addition, the downstream benefits of mindfulness-based neurofeedback require systematic examination. There is evidence for the feasibility of neurofeedback with clinical populations, and future work should directly compare the effects of mbNF between clinical and non-clinical populations.

### 4.7 Limitations

Our conclusions should be tempered in light of the heterogeneity of the studies. Targets, outcomes, and sample characteristics varied widely across the studies. These differences are well known to affect neural outcomes (e.g., neuromaturation in adolescents,Fan et al., 2021;Norbom et al., 2021). Mindfulness training may be more effective for reducing psychological distress than for improving cognitive function (Gill et al., 2020;Whitfield et al., 2022), and it is unclear whether this applies to mbNF. Reporting was also variable, which we assessed using the CRED-NF checklist (Ros et al., 2020). The vast majority of studies lacked blinded control conditions (e.g., sham neurofeedback), reported brain target engagement as a single outcome instead of comprehensively over time, and did not engage in open science practices such as preregistration. In the future, full reporting of targets and outcomes could help identify why some studies may see effects and others do not, and it could lead to possible quantitative synthesis of effects.

Another limitation is the scope of the review. We chose not to review all meditation-based neurofeedback, restricting our selection to studies that employed mindfulness practices. There are multiple families of meditations, including attentional, constructive, and deconstructive practices (Dahl et al., 2015). The studies included here involved attentional practices. Future work should examine the effects of neurofeedback on other practices, perhaps targeting different brain processes.

### 4.8 Future directions

To study the mechanisms of mbNF and associated effects, the field would benefit from adopting best practices. Chief among these may be a confirmatory–exploratory distinction. Exploratory studies may examine multiple targets, multiple modalities of feedback, qualitative as well as quantitative feedback—all with the aim of establishing preliminary hypotheses about neural targets. These studies are necessary and important given the nascency of the field. Three studies reviewed provide a sound roadmap for this type of work (Garrison, Santoyo, et al., 2013;Garrison, Scheinost, et al., 2013;Van Lutterveld et al., 2017). One innovation in particular is working with experienced meditators, who have detailed awareness of mental phenomena during meditation. Another innovation is developing individualized targets—one method could be monitoring neural data during meditation for a given participant, with self-report probes (experience samples), and then in a subsequent task delivering feedback that was trained on that initial period. This personalization may be more effective than using “one-size-fits-all” brain signals (Brandmeyer & Reggente, 2023).

It is critically important, however, to build on this work using RCTs with carefully designed sham control conditions. Such confirmatory work, through tests of clear and a priori hypotheses, can help the field evaluate whether participants learn to modulate a neural signal, and whether it leads to higher state mindfulness and positive mental health or cognitive outcomes. Sham or alternative ROI controls are preferred, given their ability to control for effects of placebo as well as feedback monitoring, but mindfulness-as-usual controls are useful and easier to implement. Researchers may even choose to examine different dosages of mbNF (Bloom et al., 2023). Clinical trial registration and/or pre-registration is useful, and when deviations emerge as they always do during empirical research, they should be reported. As mentioned previously, the CRED-NF checklist should be used for standardized reporting.

A final aim is real-world translation. In contrast to fMRI, which is costly and largely only accessible via academic medical centers, there is burgeoning interest in consumer-grade EEG tools such as MUSE (Hashemi et al., 2016;Sawangjai et al., 2019), which are relatively cheap (~$250) and easy to use. We believe that this interest should be tempered given the limited knowledge base in laboratory settings. EEG tools such as MUSE may rely on the potent influence of*neurosuggestion*(Schönenberg et al., 2017), which is a cultural emphasis and trust in Western society for neuroscientific technology. Speculatively,*neurosuggestion*effects may not be sustainable in supporting a habit of meditation, and may obscure the self-insight that comes with meditation (Vago & David, 2012).

## Supplementary Material

Supplementary Material

Supplement2_PRISMA_Checklist
