# Supplementary material for: Mindfulness-based neurofeedback: A systematic review of EEG and fMRI studies
Source: Imaging Neurosci (Camb). 2024 Dec 20;2:imag-2-00396. doi: 10.1162/imag_a_00396 (PMC12315751; doi:10.1162/imag_a_00396)
Supplement: Supplementary Material [file imag_a_00396-supp.pdf]

Supplement

Table S1: CRED-NF items, full

Domain	Item #	Checklist item
**Pre-experiment**		
	1a	Pre-register experimental protocol and planned analyses
	1b	Justify sample size
**Control groups**		
	2a	Employ control group(s) or control condition(s)
	2b	When leveraging experimental designs where a double-blind is possible, use a double-blind
	2c	Blind those who rate the outcomes, and when possible, the statisticians involved
	2d	Examine to what extent participants and experimenters remain blinded
	2e	In clinical efficacy studies, employ a standard-of-care intervention group as a benchmark for improvement
**Control measures**		
	3a	Collect data on psychosocial factors
	3b	Report whether participants were provided with a strategy
	3c	Report the strategies participants used
	3d	Report methods used for online-data processing and artifact correction
	3e	Report condition and group effects for artifacts
**Feedback specifications**		
	4a	Report how the online-feature extraction was defined
	4b	Report and justify the reinforcement schedule
	4c	Report the feedback modality and content
	4d	Collect and report all brain activity variable(s) and/or contrasts used for feedback, as displayed to experimental participants
	4e	Report the hardware and software used
**Outcome measures**		
Brain	5a	Report neurofeedback regulation success based on the feedback signal
	5b	Plot within-session and between-session regulation blocks of feedback variable(s), as well as pre-to-post resting baselines or contrasts
	5c	Statistically compare the experimental condition/group to the control condition(s)/group(s) (not only each group to baseline measures)
Behaviour	6a	Include measures of clinical or behavioural significance, defined a priori, and describe whether they were reached
	6b	Run correlational analyses between regulation success and behavioural outcomes
**Data storage**		
	7a	Upload all materials, analysis scripts, code, and raw data used for analyses, as well as final values, to an open access data repository, when feasible

Table S2: fMRI CRED-NF coding

Domain	Item #	Checklist item	Garrison2013a	Garrison2013b	Kim2019	Bauer2020	Pamplona2020	Pamplona2023	Kirlic2022	Yu2022	Zhang2023
**Pre-experiment**											
	1a	Preregistration	N	N	Y	N	N	N	Y	N	N
	1b	**Justify sample size**	N	N	N	N	N	N	Y	Y	Y
**Control groups**											
	2a	**Controls employed**	N*	Y	Y	Y	Y	N	N	N **	N
	2b	**Double-blind**	N	N	Y	N	N	N	N	N	N
	2c	Blind outcomes	N	N	Y	N	N	N	N	N	N
	2d	Assess blinding	N	N	Y	N	N	N	N	N	N
	2e	Standard-of-care control	NA	NA	NA	N	NA	NA	NA	NA	N
**Control measures**											
	3a	Psychosocial factors	N	N	Y	N	Y	Y	Y	Y	N
	3b	**Report provided strategy**	Y	Y	Y	Y	Y	Y	Y	Y	Y
	3c	Actual strategies	Y	Y	N	N	Y	Y	N	N	N
	3d	**Data processing**	Y	Y	Y	Y	Y	Y	Y	Y	Y
	3e	Artifacts by condition	Y	Y	Y	N	N	N	N	N	N
**Feedback specifications**											
	4a	**Target extraction**	Y	Y	Y	Y	Y	Y	Y	Y	Y
	4b	**Reinforcement reporting**	Y	Y	Y	N	Y	Y	N	N	N
	4c	**Modality of feedback**	Y	Y	Y	Y	Y	Y	Y	Y	Y
	4d	**Report target**	Y	Y	Y	Y	Y	Y	Y	Y	Y
	4e	**Hardware/soft**	Y	Y	Y	Y	Y	Y	Y	Y	Y
**Outcome measures**											
Brain	5a	**Report target success**	Y	N	Y	Y	Y	Y	Y	Y	Y
	5b	**Target + baseline**	Y	N	Y	N	Y	Y	Y	N	N
	5c	**Control vs feedback**	Y	N	Y	N	N	N	N	N	N
Behaviour	6a	**Behavioral outcomes**	N	N	Y	Y	Y	Y	Y	N	Y
	6b	**Correlation to target**	N	N	Y	Y	Y	N	Y	N	Y
**Data storage**											
	7a	Open science	N	N	N	N	Y	Y	N	N	N

Bold items reflect essential checklist items. Please note that Garrison2013a and Garrison 2013b used same protocol, Pamplona2020 and Pamplona2023 used same sample, and Kirlic2022 and Yu2022 used same sample. This means we coded ‘Y’ for those studies if either manuscript reported a methodological detail (but not for outcomes). NA: not applicable, in the case of non-clinical samples,

there is no ‘standard-of-care’. Note that Kirlic2022 and Yu2022 filled out checklists, which we re-coded where necessary for this review. * Garrison2013a reported non-meditators as a control but they also received mbNF. ** Yu2022 reported controls in their checklist, but their controls are not between-subject or within-subject, instead they are just baseline resting-state.

Table S3: EEG CRED-NF coding

Domain	Item #	Checklist item	Hinterberger2016	Kosunen2016	Salminen2023	vanLutterveld2017	Dunham2018	Dunham2019	Prestel2019	Brandmeyer2020	Chen2021
**Pre-experiment**											
	1a	Preregistration	N	N	N	N	Y	Y	N	N	N
	1b	**Justify sample size**	N	N	N	Y	N	N	N	N	N
**Control groups**											
	2a	**Controls employed**	Y	Y	Y	Y	N	N	Y	Y	Y
	2b	**Double-blind**	N	N	Y	N	N	N	Y	N	N
	2c	Blind outcomes	N	N	N	N	N	N	N	N	N
	2d	Assess blinding	N	N	N	N	N	N	N	N	N
	2e	Standard-of-care control	NA	NA	NA	NA	NA	NA	NA	N	N
**Control measures**											
	3a	Psychosocial factors	Y	Y	Y	N	N	Y	Y	Y	N
	3b	**Report provided strategy**	Y	Y	Y	Y	Y	Y	Y	Y	Y
	3c	Actual strategies	N	N	Y	N	N	Y	N	N	N
	3d	**Data processing**	N	N	Y	N	N	Y	Y	Y	Y
	3e	Artifacts by condition	N	N	Y	N	N	N	N	N	N
**Feedback specifications**											
	4a	**Target extraction**	Y	Y	Y	Y	Y	Y	Y	Y	Y
	4b	**Reinforcement reporting**	Y	Y	Y	N	Y	Y	Y	N	N
	4c	**Modality of feedback**	Y	Y	Y	Y	Y	Y	Y	Y	Y
	4d	**Report target**	Y	Y	Y	N	N	Y	Y	Y	Y
	4e	**Hardware/soft**	Y	Y	Y	Y	Y	Y	Y	Y	Y
**Outcome Measures**											
Brain	5a	**Report target success**	Y	N	Y	Y	Y	Y	Y	Y	N
	5b	**Target + baseline**	Y	N	N	N	N	N	Y	Y	N
	5c	**Control vs feedback**	Y	Y	Y	Y	N	N	N	Y	Y
Behaviour	6a	**Behavioral outcomes**	N	N	N	N	N	N	N	N	N
	6b	**Correlation to target**	N	N	N	Y	N	N	N	N	N
**Data storage**											
	7a	Open science	N	N	N	N	N	N	N	Y	N

Bold items reflect essential checklist items. Hinterberger2016: Hinterberger & Funrohr 2016, Kosunen2016: Kosunen et al., 2016, Salminen2023: Salminen et al., 2023, vanLutterveld2017: van Lutterveld et al., 2017, Dunham2018: Dunham et al., 2018, Dunham2019: Dunham et al., 2019, Prestel2019: Prestel et al., 2019, Brandmeyer2020: Brandmeyer & Delorme, 2020, Chen: Chen et al., 2021 etc.

Table S4: fMRI Qualitative Ratings

Garrison, Scheinost, et al., 2013	Exp 1: After each run, participants rated how well the graph corresponded with their moment-to-moment subjective experience, briefly described this rating, and rated how well they were able to follow instructions.<br>Exp 2: After each run, participants described their experience during focused attention meditation, rated how well the graph corresponded with experiencing during meditation (for both offline and real-time feedback), and reported what strategy they used to decrease the feedback graph.
Garrison, Santoyo, et al., 2013	Same as Exp 2 in Garrison, Scheinost, et al., 2013
Kim et al., 2019	Not reported
Bauer et al., 2020	Not reported
Pamplona et al., 2020	After each NF run, participants rated their control over the thermometer, how difficult it was to control the thermometer, and their concentration level.
Pamplona et al., 2023	After each NF run, participants rated what strategy they used and their concentration level.
Kirlic et al., 2022	After each run, participants reported how well they were able to follow instructions on the screen, how easy they found it to focus on breath, how much their mind wandered, how easy it was to mentally decide whether or not words described them, how easy it was to clear their mind while resting, and how they felt.<br>After each NF run, participants reported how well the blue bar corresponded with their experience of breath focus, and how well the red bar corresponded with their experience of mind wandering.
Yu et al., 2022	Same as Kirlic et al., 2022
Zhang et al., 2023	After each run, participants confirmed that they had used mental noting during NF

Table S5: EEG Qualitative Ratings

Hinterberger & Fürnrohr, 2016	Participants completed a feedback questionnaire after each interventional condition, rating their physical sensation, emotional condition, mental state, experience, motivation, duration, and relevance. Participants were also asked, "Could you notice a connection between yourself and the audiovisual perceptions?"
Kosunen et al., 2016	After each experiment condition, participants completed the ITC-Sense of Presence Inventory and a meditation depth questionnaire measuring five factors: Hindrance, Relaxation, Personal Self, Transpersonal Qualities, and Transpersonal Self.

Salminen et al., 2023	After each experiment condition, participants completed the ITC-Sense of Presence Inventory and the 6-item factor 'Hindrances' from a meditation depth questionnaire.
van Lutterveld et al., 2017	Participants were asked which direction of the graph they associated with their subjective experience of effortless awareness, and rated their confidence in their response.
Dunham et al., 2018	Not reported
Dunham et al., 2019	After session 2 of each learning day, participants rated their perceptions that the following cognitive states were associated with reductions in the BIS values: widening the visual field, decreasing effort, attention to space, and relaxed alertness.
Prestel et al., 2019	Participants completed a semi-structured interview immediately after each NF session, reporting on their perceived success in changing the signal, which strategies they applied during each block, difficulties or distractions, and an overall evaluation of the session.
Brandmeyer & Delorme, 2020	Participants reported on whether they were able to successfully implement one of the learned strategies after each day of NF.
Chen et al., 2021	Not reported

Figure S1: Sample sizes for unique samples

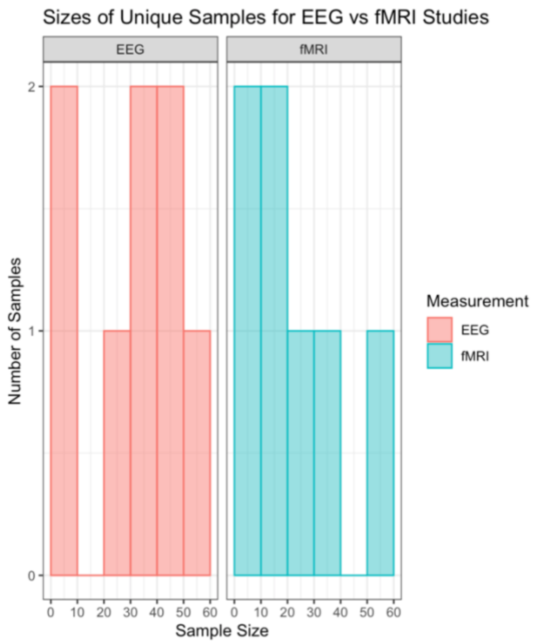

Figure S1. Side by side histograms comparing the unique sample sizes of EEG studies and fMRI studies. Duplicate samples are excluded, meaning that in cases where the same sample of participants resulted in multiple reports, only one sample was counted. In cases where duplicate samples have different sample sizes, the higher sample size was included.

Figure S2: Types of control conditions

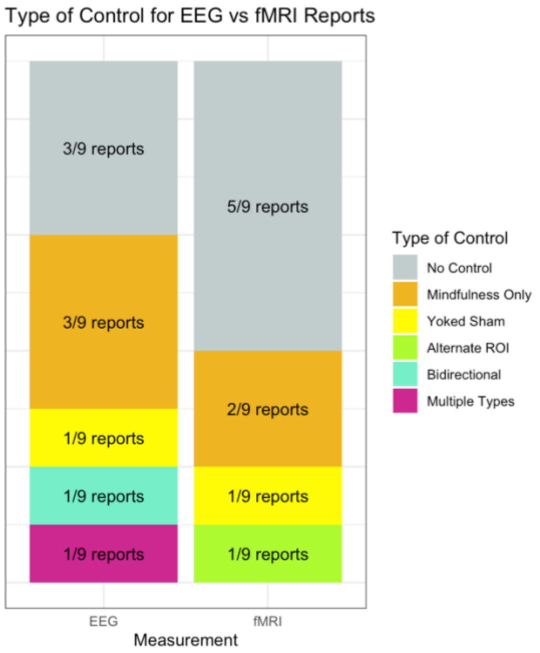

Figure S2. Stacked bar chart showing the types of control conditions in EEG and fMRI reports. Reports that included more than one type of control condition are counted as 'multiple types.' A higher proportion of EEG reports have some sort of control, largely driven by 'mindfulness only' control conditions. On the other hand, there are alternate ROI controls in the fMRI studies which are not present in EEG. Some experiments had multiple conditions of the same control type (for example, two 'mindfulness only' conditions with different types of mindfulness performed).

Figure S3: Types of control conditions, within-person vs between-person

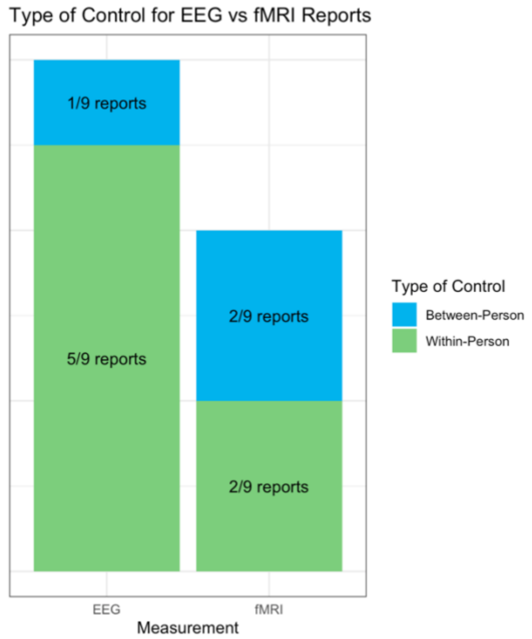

Figure S3. For reports with control conditions, this stacked bar chart shows the quantities of between-person and within-person controls for EEG and fMRI studies. EEG studies are predominantly within-person, whereas fMRI include between-person and within-person controls equally.
